# Costs of early detection systems for epidemic malaria in highland areas of Kenya and Uganda

**DOI:** 10.1186/1475-2875-8-17

**Published:** 2009-01-16

**Authors:** Dirk H Mueller, Tarekegn A Abeku, Michael Okia, Beth Rapuoda, Jonathan Cox

**Affiliations:** 1 Health Economics and Financing Programme, Department of Public Health and Policy, London School of Hygiene & Tropical Medicine, Keppel St., London WC1E 7HT, UK; 2 Disease Control and Vector Biology Unit, Department of Infectious and Tropical Diseases, London School of Hygiene & Tropical Medicine, Keppel St., London WC1E 7HT, UK; 3 National Malaria Control Programme, Ministry of Health, Uganda; 4 Deceased author; formerly Division of Malaria Control, Ministry of Health, Kenya

## Abstract

**Background:**

Malaria epidemics cause substantial morbidity and mortality in highland areas of Africa. The costs of detecting and controlling these epidemics have not been explored adequately in the past. This study presents the costs of establishing and running an early detection system (EDS) for epidemic malaria in four districts in the highlands of Kenya and Uganda.

**Methods:**

An economic costing was carried out from the health service provider's perspective in both countries. Staff time for data entry and processing, as well as supervising and coordinating EDS activities at district and national levels was recorded and associated opportunity costs estimated. A threshold analysis was carried out to determine the number of DALYs or deaths that would need to be averted in order for the EDS to be considered cost-effective.

**Results:**

The total costs of the EDS per district per year ranged between US$ 14,439 and 15,512. Salaries were identified as major cost-drivers, although their relative contribution to overall costs varied by country. Costs of relaying surveillance data between facilities and district offices (typically by hand) were also substantial. Data from Uganda indicated that 4% or more of overall costs could potentially be saved by switching to data transfer via mobile phones. Based on commonly used thresholds, 96 DALYs in Uganda and 103 DALYs in Kenya would need to be averted annually in each district for the EDS to be considered cost-effective.

**Conclusion:**

Results from this analysis suggest that EDS are likely to be cost-effective. Further studies that include the costs and effects of the health systems' reaction prompted by EDS will need to be undertaken in order to obtain comprehensive cost-effectiveness estimates.

## Background

Despite continuing research on the epidemiology and control of malaria epidemics, little is known about the public health burden associated with these events [1]. The data that are available indicate that epidemics can cause widespread morbidity, and that epidemic-related risks of severe disease and death are relatively high across all age groups affected [2-4]. Moreover, little is known about the economic burden of epidemics, or the costs of interventions used for epidemic prevention and control. Without reliable information in this area policy makers are unable to make informed resource allocation decisions based on sound evidence [5].

For many years, the World Health Organization (WHO) has advocated the development and use of malaria early warning systems (MEWS) in areas of unstable malaria transmission [6,7]. Although no standard, single model for MEWS exists, it is generally accepted that MEWS may incorporate a variety of information sources, including seasonal climate forecasts, observed meteorological data and monitored malaria case loads. A distinction is often made between two types of systems: those, which try to predict future malaria incidence (epidemic 'forecasting', 'early warning' or 'prediction' systems), and, others, which use malaria surveillance data to detect epidemics very early in their progression ('early detection systems'). In principle, epidemic prediction systems should be able to provide warnings several weeks before the onset of an epidemic and thereby facilitate the implementation of epidemic prevention measures. However, the technical feasibility of prediction systems is still debated and their likelihood of success is expected to be context specific [8-11]. More generally, the rationale for developing sophisticated predictive models in settings where basic malaria case surveillance is weak or absent has been questioned [8]. In these instances, strengthening existing modes of malaria surveillance to enable epidemic early detection is an appropriate first step in improving the management of epidemics.

Key characteristics of epidemics, including their causality, presentation, evolution and impact, vary markedly between epidemic-prone settings and over time at individual localities [3,12]. In practice, the period within which meaningful intervention is possible (i.e. the period between an epidemic becoming 'detectable' and its subsequent peak) will vary between epidemics. The purpose of an early detection system (EDS) is to maximize the size of this operational window and thereby to give response measures the best chance of being effective. An EDS can, therefore, be seen as a prerequisite for effective epidemic response; however, in reality, the usefulness of an EDS will also be determined by the timeliness of the malaria control response once an alert has been made. An EDS can only be effective as part of a broader epidemic management strategy which includes an appropriate and specific epidemic preparedness plan [13].

As part of the Highland Malaria Project (HIMAL) an EDS was developed and piloted between 2002 and 2006 in North Nandi and Gucha districts in Kenya, and Rukungiri and Kabale districts in Uganda. These districts are located in highland or highland fringe areas and are prone to malaria epidemics to varying degrees [14]. The district population ranges from 295,000 in Rukungiri to 537,000 in Gucha (2006 estimates, Kenya Central Bureau of Statistics and Uganda Bureau of Statistics). Both countries' health systems are widely decentralized to District Health Offices and District Health Management Teams (DHMTs) in their operations, planning and budgeting.

The HIMAL EDS comprised 20 sentinel health facilities (five health centres in each of the four pilot districts), chosen to represent a variety of transmission settings across the four districts. The EDS was based on existing surveillance structures and was designed to streamline the flow of malaria data between peripheral health facilities, DHMTs and the Ministry of Health (MOH). The aim was to make analysis and interpretation of incoming surveillance data more timely by shifting primary responsibility for these activities from the MOH to the district level. Within the EDS representative sentinel sites reported daily tallies of malaria cases (by age group and administrative unit) on a weekly basis to DHMTs, where the data were entered and analysed using a customized computer database application. This database was used to produce reports based on an automated assessment of current morbidity levels against de-seasonalized and de-trended historical data [14].

Implementation of the EDS required software development and provision of capital equipment such as computers and associated peripherals for data management and dissemination, motorcycles for supervision of sentinel sites by DHMT staff and vehicles for supervision of district-level activities by MOH staff. Extensive training of selected DHMT staff, particularly in data management, was carried out and health facility staff received refresher training on malaria diagnosis, record keeping and reporting. A more detailed description of logistical and financial inputs provided by the HIMAL project is described by Jones *et al *[15].

The purpose of the analysis reported in this paper is to estimate the capital and recurrent costs associated with the setting up and routine operation of the HIMAL EDS and to provide estimates of the number of Disability-Adjusted Life Years (DALYs) that would need to be averted for the system to be cost-effective.

## Methods

The economic costs of the intervention, including full opportunity costs (such as staff time and donations [16]), were assessed from the provider's perspective, i.e. costs that would be covered by the public health care system but not the beneficiaries. In addition to the development and piloting of an EDS, HIMAL also included a number of other research elements (relating to the collection of parasitological, entomological and meteorological data; further details are presented by Abeku *et al*. [14]). For the purposes of this paper, costs were divided between implementation and research activities and all research related costs (including salary top-ups) are excluded from the analysis. However, external (international) technical assistance is considered in the sensitivity analysis.

Costs were assessed by means of the ingredients approach (i.e. by collecting expenditure of all 'ingredients' necessary to perform a particular intervention rather than using aggregate costs [17]) and categorized into capital and recurrent costs. Recurrent costs included primarily opportunity costs of staff time, expenditure on consumables and travel, and overhead costs. Capital costs included vehicles needed for supervision at national and district levels, as well as computers, printers and scanners. Costs were also differentiated between setup costs and running costs, whereby setup costs included training of staff and the compilation of retrospective malaria data.

For purchases and recorded financial transactions, costs were assessed through expenditure reviews. To estimate time spent on EDS-specific tasks and the opportunity costs in terms of apportioned salary payments, all staff involved in the EDS were interviewed. Where possible, observations of the time needed to perform the weekly compilation of malaria case data was observed at individual facilities (when the staff was already well adept in the use of the forms).

All costs were calculated based on 2006 prices. Expenditures in earlier years were inflated to 2006 prices (using inflation rates stated by the International Monetary Fund [18]). Average exchange rates were applied from the start of the EDS in October 2002 until 2006; 1 US Dollar (US$) equals 77.28 Kenya Shillings (KSh) and 1,805.67 Uganda Shillings (USh) (Oanda Corporation). All capital expenditures were annualized across their useful lifetime at a discount rate of 5%. Other set-up costs for training were spread over the project duration to obtain annual average costs.

The costing was strictly limited to the EDS only. Costs associated with response measures implemented on the basis of epidemic alerts were not included in this study. As such, the primary aim of the study is to estimate the incremental costs needed to set up and run an EDS on top of a functioning health care system.

Due to the inherent and intractable challenges involved in measuring the incremental effect of the EDS, a threshold analysis was carried out to determine the number of DALYs that would need to be averted for the EDS to be cost-effective. Thresholds of US$ 30 and US$ 150 per DALY have been recommended as a basis for considering an intervention either highly cost-effective or cost-effective, respectively [19].

A sensitivity analysis was performed to model potential variations in the costs to the EDS, such as the inclusion of external technical assistance, a 10% increase in salary of health staff, changes in the discount rate from 5% to 3% and 7% and changes to the exchange rates to the highest annual average and the lowest annual average during the period of operation between October 2002 and February (Kenya) and April (Uganda) 2006.

During the course of the study, it became apparent that a major amount of staff time was spent transporting surveillance data from facility level to the district health office – typically using public transport. Consequently, for the Ugandan health facilities, opportunity costs of staff time were differentiated between data compilation at the health centre and the subsequent transport of data from the health centre to the DHMT. This distinction made it possible to model the potential cost savings that could be achieved by using more efficient means of data transfer (e.g. electronic transfer of data by mobile telephone text message).

## Results

The annual costs of the EDS were estimated at US$ 14,439 per district in Uganda and US$ 15,512 per district in Kenya. Depending on the population of each district, this translates into costs of US$ 0.03–0.05 per annum per head of population in Uganda and US$ 0.03–0.04 in Kenya. 34% of total costs were related to setup activities, such as training, purchase of equipment and vehicles, while 66% represented running costs, principally in the form of expenditure on salaries and transport. Across all sites, capital and recurrent expenditures constituted 26% and 74% of total costs respectively.

The annual costs for both countries, disaggregated by different line items, are presented in Table 1. While staff was not specifically hired for EDS (with the exception of research related staff which is excluded here), the time spent on EDS by each member of staff was estimated as part of the economic costing (Table 2). This demonstrated that staff time invested in EDS was substantial at all levels of staff involved and salaries were therefore the most important cost drivers in both countries, albeit to varying degrees. In Kenya, salaries made up 56% of overall costs per annum, while in Uganda this figure was 41%. The distribution of salary costs between different levels of the health system also varied between the two countries. In Uganda the largest proportion of salaries was used to support staff at the peripheral (health centre) level; in Kenya the largest proportion of salaries was related to national level supervision. These differences reflect both differing salary structures between the two countries and differences in the amount of reported time invested by personnel at national level.

**Table 1:** Annual economic costs of running the early detection system per district in both countries in US$ (based on 2006 prices)

**Line item category**	**Uganda per district (US$)**	**Kenya per district (US$)**	**% Uganda**	**% Kenya**
**Equipment**	**3,947**	**4,015**	**27.3**	**25.9**
**Consumables**	**502**	**91**	**3.5**	**0.6**
Salaries	5,860	8,753	40.6	56.4
Per diems	20	84	0.1	0.5
**Total personnel**	**5,880**	**8,837**	**40.7**	**57.0**
Fuel	1,426	316	9.9	2.0
Maintenance	274	155	1.9	1.0
**Total transport (excl. training)**	**1,700**	**472**	**11.8**	**3.0**
**Travel & accommodation (for training)**	**590**	**64**	**4.1**	**0.4**
**Utilities**	**623**	**797**	**4.3**	**5.1**
**Room rental**	**521**	**816**	**3.6**	**5.3**
**Fees**	**93**	**19**	**0.6**	**0.1**
**Other**	**585**	**401**	**4.1**	**2.6**

**TOTAL**	**14,439**	**15,512**	**100.0**	**100.0**

**Table 2:** Reported percent of full-time equivalent spent by each member of staff on EDS (by staff position)

	**% of time spent on EDS (average)**	
**Position**	**Kenya**	**Uganda**
**National level (MOH)**		
Head of Programme	15%	5%
Parasitologist	50%	23%
Support staff	51%	35%
**District level (DHO)**		
District Medical Officer	1%	1%
District Public Health Officer	19%	22%
District Surveillance Officer	6%	24%
**Health facility level**		
Clinical Officer in-charge	4%	7%
Records Assistant	15%	16%

Other important cost drivers were related to equipment (vehicles and computers) accounting for 27% and 26% of total costs in Uganda and Kenya, respectively. Transport related costs amounted to 12% in Uganda, but only 4% in Kenya. Rental and utilities of office space added to 8% in Uganda and 10% in Kenya.

In Uganda opportunity costs of staff time spent on transporting data were assessed specifically. These added up to US$ 1,125 per year across the ten facilities in both districts (representing more than 7% of overall costs). Adopting an electronic system of data transfer (using e.g. mobile telephone text message) could potentially reduce these costs by more than half, even if all facilities sent several messages each week (cost estimated at US$ 1 per week per facility).

The sensitivity analysis (presented in Table 3) showed that a salary increase of 10% within both countries would change the annual costs per district by 4.1% in Uganda (from US$ 14,439 to US$ 15,025) and by 5.6% in Kenya (from US$ 15,512 to US$ 16,387). Changes in the exchange rates to a lower value of the US Dollar to the two currencies (one US$ equalling USh 1,737.85 and KSh 72.62) showed an increase in the annual costs per district by 4.2% in Uganda (to US$ 15,042) and by 6.3% in Kenya (to US$ 16,484). Changes to a higher value of the US Dollar compared to the Kenyan Shilling and the Ugandan Shilling (one US$ equalling USh 1,846.83 and KSh 79.55) lowered annual costs per district by 4.1% in Uganda (to US$ 13,842) and by 4.5% in Kenya (to US$ 14,807).

**Table 3:** Sensitivity analysis – impact of varying assumptions on total costs (US$)

**Variable**	**Change**	**Impact on annual costs per district in Uganda**	**Impact on annual costs per district in Kenya**
Salary increase	10%	Increase from US$ 14,439 to US$ 15,024.	Increase from US$ 15,512 to US$ 16,387.
Exchange rate: US$ 1 =	USh 1,737.85 KSh 72.62	Increase from US$ 14,439 to US$ 15,042.	Increase from US$ 15,512 to US$ 16,484.
Exchange rate: US$ 1 =	USh 1,846.83 KSh 79.55	Decrease from US$ 14,439 to US$ 13,842.	Decrease from US$ 15,512 to US$ 14,807.
Discount Rate	From 5% to 7%	Increase from US$ 14,439 to US$ 14,653.	Increase from US$ 15,512 to US$ 15,728.
Discount Rate	From 5% to 3%	Decrease from US$ 14,439 to US$ 14,229.	Decrease from US$ 15,512 to US$ 15,300.
Relay of malaria surveillance data from facility	Changes from "In person" to "wireless"	Decrease from US$ 14,439 to US$ 13,834.	Not tested.

Assuming that no external technical assistance is provided, the EDS would need to avert annually 96 and 103 DALYs per district in Uganda and Kenya, respectively, for it to be considered cost effective at a threshold of US$ 150 per DALY averted (not considering the health system's response). Similarly, to be considered highly cost-effective (at a threshold of US$ 30 per DALY averted), the EDS would need to prevent annually 481 and 517 DALYs per district, respectively. Given that most malaria related DALYs are made up of fatal cases [20], the EDS would need to avert roughly 5 deaths annually per district in either country to make it cost-effective, or 24–26 deaths for it to be considered highly cost effective.

## Discussion

Epidemic-prone areas present a number of challenges to malaria control. Low levels of immunity to malaria in local populations means that clinical attack rates during epidemic events can be extremely high [2]. Once infected, people of all ages are at relatively high risk of developing severe forms of malaria (including cerebral malaria) and case fatality rates associated with epidemics are often very high [3,4,21]. During non-epidemic periods, however, prevailing rates of malaria transmission are low, which means that routine deployment of preventative measures such as IRS is not usually justified, and that uptake of some interventions (e.g. ITNs) at the community level may be limited. For these reasons, malaria control in unstable transmission settings is typically reactive, and relies on precise deployment of interventions in space and time. In practice, however, effective targeting of this type has rarely been achieved, primarily because standard modes of surveillance offer insufficient time to organize a coherent response.

The purpose of MEWS is to improve the management of major epidemics as well as minor outbreaks through increasing the time period during which control (or possibly preventive) measures can be deployed. In theory, epidemic prediction systems based on seasonal climate forecasts or observed climate variables may be able to provide lead times of several months or weeks, and thereby provide a relatively long period of time during which control operations can be planned and implemented. To date, however, operational research in this area has been limited and the complexity of malaria transmission systems in many epidemic-prone settings in highland areas may make the development of such systems difficult to achieve in the short-term [8].

In the absence of epidemic prediction, MEWS may still contribute to improved epidemic management by bringing forward the point at which epidemics are detected and declared [14,22]. Various algorithms for epidemic detection have been developed and applied [11], but broader questions around the cost and sustainability of EDS have received relatively little attention.

This study estimated the economic costs per year per district needed to run such an EDS based upon enhanced surveillance. The total costs ranged between US$ 14,400 and US$ 15,500, depending on the country and district. Costs per head of population varied between US$ 0.03 and US$ 0.05. The comparison between countries and the sensitivity analysis of exchange, discount rates and salary increases demonstrate the robustness of the data (Table 3). It is important to note that the figures presented here represent economic costs including all opportunity costs of resources used (in particular personnel). Financial costs would differ substantially depending on whether salaries of facility, district and national level staff were included or not as the time spent on EDS by each member of staff was substantial at all levels (Table 2). Personnel costs account for the largest component of economic costs (41% in Uganda and 57% in Kenya).

Most of these costs are not expected to be subject to economies of scale should more districts or larger areas be incorporated into the EDS. However, savings could be achieved by increasing the efficiency of the data transfer process. In Uganda, for example, using mobile telephones to relay health facility data to DHMTs could potentially reduce the opportunity costs of personnel time (by more than 4% of total costs).

In the case of HIMAL, the development of district-level EDS (led by local stakeholders) represented a direct response to demand for new tools for epidemic management on the part of East African malaria control programmes (see annexe in ref. [23]). Although it is likely that the availability of external technical assistance was an important catalyst for the development and implementation of the EDS in both countries, experience within HIMAL also suggests that the system can function without external assistance [14]. In Kenya, plans have been drawn up to extend EDS activities to other epidemic-prone districts, using the HIMAL EDS system as a model. For this and similar programmes, the costs presented in the current analysis (and which exclude external technical support) are likely to provide a realistic indication of the scale of resources needed to establish and maintain a district-based EDS.

However, were costs of external support to be included, overall annual costs per district would more than double, to US$ 34,039 in Uganda and US$ 35,113 in Kenya. These estimates should, however, be interpreted with caution. The current exercise tried to distinguish (and exclude) costs relating to HIMAL research activities; but in practice the distinction between research and technical support is subject to individual interpretation. Moreover, costs for external technical assistance may well have been different had support for surveillance activities been provided by an international development agency or NGO.

It is also important to consider whether interventions such as EDS, which incorporate new activities and areas of responsibility into an existing health system, can be expected to operate effectively in the absence of additional incentives to the staff. Interviews carried out with 52 health staff at district and central level revealed varying opinions on the necessity of top-ups for sustaining the EDS [15]. Most respondents felt that some form of remuneration was necessary to cover out of pocket expenses (e.g. relating to travel), but many also stated that availability of resources to cover basic surveillance activities (e.g. fuel and vehicles for supervision, stationary) was the most critical determinant of the success or failure of the system [15]. Staff also frequently cited a number of non-pecuniary factors that contributed to their motivation – including increased self-efficacy, responsibility and recognition [15]. In the current study we took account of all operational costs, including expenditure for local travel, as well as the opportunity cost of staff. However, motivational (financial) incentives were excluded in this costing, as the EDS is expected to continue without these incentives. In reality, the validity of such an assumption is likely to vary between different settings and programmes.

An EDS is, by definition, a complementary step to facilitate a timely range of activities by the health system to limit the malaria case load. This includes contingency planning and community liaison, as well as direct intervention through improved case management, and, where appropriate, mass drug administration or mass fever treatment. If time allows and the abnormal transmission is expected to continue for some time, IRS is likely to be the method of choice for vector control [24-26].

In order to assess the cost-effectiveness of the EDS, the costs and effects of both the EDS and any response would ideally need to be taken into account for a comprehensive cost-effectiveness analysis. Methodologically, however, this poses various challenges [5]. Firstly, various definitions and thresholds exist to describe epidemics [3,5,11]. Secondly, to measure effectiveness, it is necessary to distinguish between epidemic-related mortality and background fluctuations in overall mortality. In practice very few community-based estimates of epidemic-related mortality exist – and these suggest that burden associated with individual epidemic events is highly variable [3]. Thirdly, some epidemic response measures (e.g. case management at health facilities) overlap with routine malaria control interventions – although epidemic-specific measures are often employed in large-scale outbreaks (such as mobile mass treatment services). Lastly, the pattern of epidemics may vary substantially between locations – and over time at a single location – which renders comparisons between different interventions (or with a control area) difficult. In addition, a study design that aims to compare malaria outcomes between intervention and control areas may be difficult to justify ethically. For the above reasons, attribution of a difference in malaria cases or deaths to an EDS and associated response measures is difficult. Even if a cost-effectiveness analysis could be carried out successfully, the context-specific nature of epidemics poses severe challenges to its transferability to other settings and regions [5].

For an approximation of the scope of epidemic malaria in the districts under study, excess cases occurring above the HIMAL epidemic detection threshold [14] were estimated, i.e. total cases during epidemic episodes minus total expected cases based on baseline observations. The estimated number of average excess cases per annum per district ranged between 9,800 – 32,000 (18 – 67 per 1,000 population).

A threshold analysis was performed to determine how many DALYs would need to be averted to consider EDS cost effective. The results of this study demonstrate that for EDS to be considered cost effective (with a cost per DALY of US$ 150 or less), roughly 100 malaria associated DALYs would need to be averted annually in each district (with populations ranging from 300,000 in Rukungiri to 550,000 in Gucha). Thus, the number of DALYs to be averted to make EDS cost effective seems comparatively small, even if costs for treatment and/or vector control are added to the costs of EDS. IRS, for example, is estimated to cost between US$ 5 and US$ 34 per DALY averted; ITN distribution would cost similarly between US$ 5 and US$ 31 per DALY averted [27]. If costs of the response would be estimated at US$ 34 per DALY averted and thus subtracted from the threshold of US$ 150 to determine a cost-effective intervention, roughly 125 DALYs would need to be averted in order to render EDS cost-effective (at a threshold of US$ 150 per DALY).

In comparison to other malaria control interventions, the incremental cost of EDS is likely to be an attractive investment in this and comparable epidemic settings. If costs and effects were analysed from a societal perspective, cost-effectiveness ratios are likely to be even more favourable as costs to the community would be minimal and epidemic malaria usually affects the working population more than malaria does in endemic areas [4].

## Conclusion

This study suggests that EDS is likely to be cost-effective. Further studies that include the costs and effects of the health system's reaction prompted by EDS will need to be undertaken in order to obtain comprehensive cost-effectiveness estimates. Further substantial savings to the overall costs of EDS are possible by transmitting surveillance data electronically rather than in person and public-private partnerships with mobile network operators could be sought to harnessing the savings and speed inherent in electronic transmission of surveillance data.

## Competing interests

The authors declare that they have no competing interests.

## Authors' contributions

DM conceptualized the costing, analysed cost data and drafted the manuscript. JC and TAA conceived and designed the EDS and revised the draft manuscript. DM, MO, BR compiled cost data on national and district level. TAA calculated estimates of potentially preventable cases and DM modelled cost-effectiveness estimates. All authors (except BR who sadly passed away) read and approved the final manuscript.
